# Tissue-specific peripheral sensory remodeling in chronic low back pain: established and emerging nociceptive niches

**DOI:** 10.3389/fnmol.2026.1972635

**Published:** 2026-10-05

**Authors:** Yizhuan Huang, Han Wang

**Affiliations:** Affiliated Sports Hospital of Chengdu Sport University, Chengdu, Sichuan, China

**Keywords:** chronic low back pain, dorsal root ganglion, intervertebral disc, neuroimmune crosstalk, nociceptive niche, peripheral sensory remodeling, thoracolumbar fascia, vertebral endplate

## Abstract

Chronic low back pain arises from heterogeneous peripheral and central mechanisms, yet the sensory biology of individual lumbar tissues is rarely considered within a unified framework. This Mini Review compares peripheral sensory remodeling and sensitization across four candidate nociceptive niches: the intervertebral disc, vertebral endplate, thoracolumbar fascia, and paraspinal muscle. Disc pathology is characterized by nociceptive ingrowth, endplate pathology by osteochondral sensory remodeling and afferent sensitization, and thoracolumbar fascia by inflammation-associated remodeling of a pre-existing sensory network. Paraspinal muscle remains an emerging sensory–inflammatory niche in which muscle-spindle remodeling is distinct from unproven nociceptor remodeling. Across tissues, recurrent neurotrophic, inflammatory, neuropeptide, and mechanosensory mediators indicate molecular overlap but do not establish functional convergence within common sensory circuits. Dorsal root ganglia and the spinal dorsal horn are therefore considered candidate sites of downstream integration. We propose a dynamic multi-niche model with testable predictions for identifying active niches, tracking shifts in tissue contribution, and distinguishing causal nociceptive mechanisms from incidental degeneration. Future spatial, longitudinal, and epigenomic studies may enable molecular definition of active pain-generating states and mechanism-guided treatment.

## 1 Introduction

Chronic low back pain (CLBP) is biologically heterogeneous and cannot be reduced to a single anatomical lesion or pain pathway. Several lumbar tissues—including the intervertebral disc, vertebral endplate, thoracolumbar fascia (TLF), and paraspinal muscle—can generate nociceptive input, yet these compartments differ markedly in baseline innervation, cellular composition, extracellular environment, and responses to mechanical and inflammatory stress. Peripheral pain generation in CLBP may therefore be more usefully conceptualized as a set of tissue-specific nociceptive niches rather than as a uniform process.

Here, peripheral sensory remodeling is used as an umbrella term for structural or persistent phenotypic changes involving sensory afferents or sensory end organs, including axonal ingrowth, redistribution of sensory fibers, altered neurochemical phenotypes, and structural remodeling of sensory end organs. We distinguish remodeling from sensitization, which primarily refers to increased afferent responsiveness or excitability. We use nociceptive niche to describe a local tissue microenvironment in which sensory afferents interact with resident, immune, vascular, or skeletal cells in ways that generate or sustain nociceptive input. This term is intended as a conceptual framework rather than a validated clinical diagnostic category.

The four lumbar compartments considered here illustrate distinct forms of peripheral sensory pathology. Disc degeneration can permit nociceptive fibers to extend into normally sparsely innervated inner regions (Freemont et al., 1997, 2002). Vertebral endplate pathology affects an already innervated osteochondral compartment, with osteoclast-derived Netrin-1 and Piezo2–calcitonin gene-related peptide (CGRP) signaling providing mechanistic links between tissue remodeling and nociception (Ni et al., 2019; Xu et al., 2026). The TLF contains a pre-existing sensory network that can undergo inflammatory sensitization and afferent redistribution (Tesarz et al., 2011; Hoheisel et al., 2015). Paraspinal muscle shows inflammatory–fibrotic pathology and structural remodeling of proprioceptive muscle spindles, although persistent nociceptor remodeling in human CLBP remains insufficiently established (Shahidi et al., 2017; James et al., 2022).

This Mini Review compares these tissue-specific sensory phenotypes and examines overlapping molecular signals and candidate sites of downstream sensory-circuit integration. Figure 1 distinguishes molecular overlap from unproven circuit convergence, while Table 1 separately appraises human observations, experimental causality, and pain-related outcomes. We also develop testable predictions for distinguishing active nociceptive niches from incidental degeneration.

**Figure 1:**
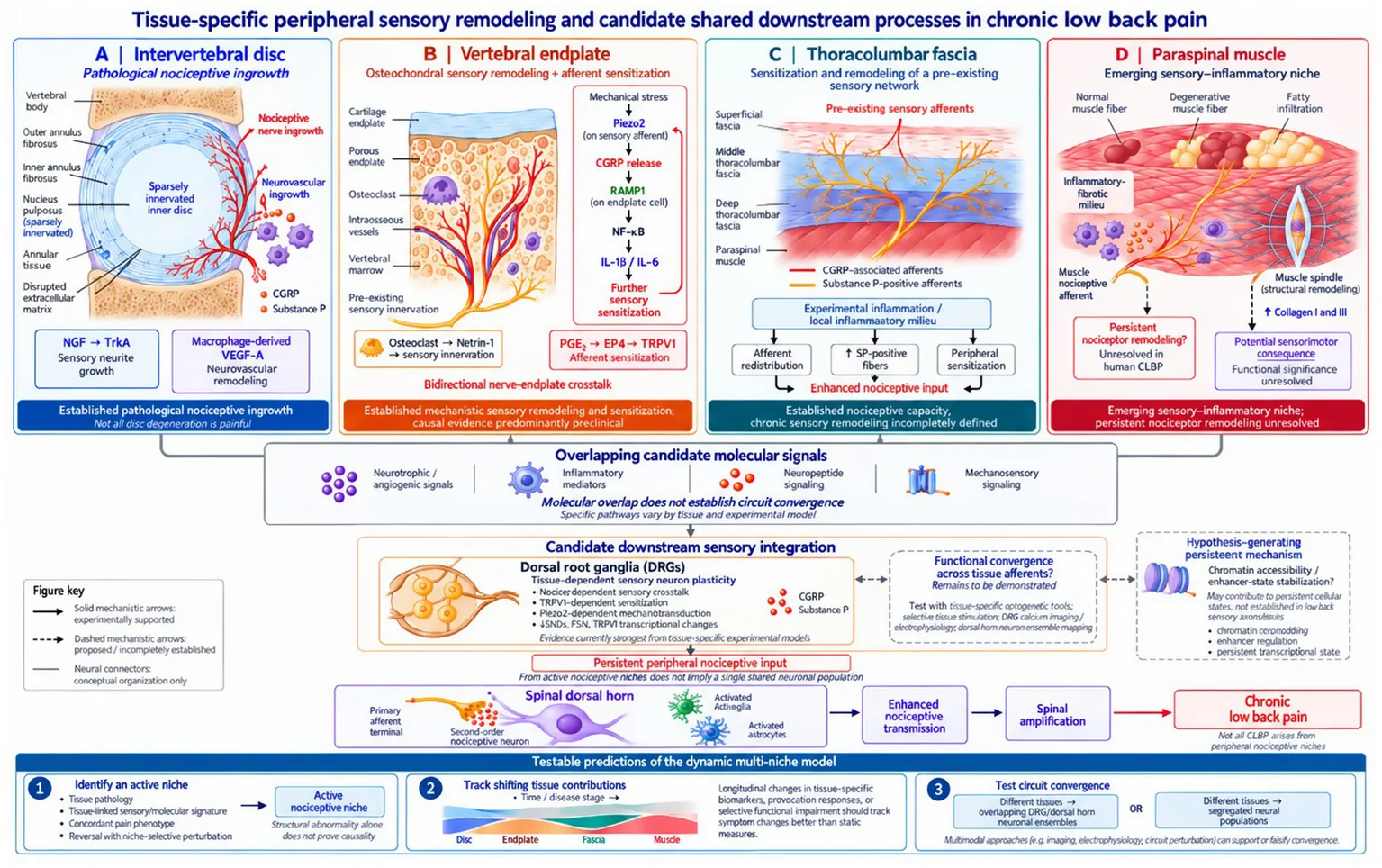
Tissue-specific peripheral sensory remodeling, overlapping molecular signals, and candidate downstream integration in chronic low back pain. The intervertebral disc, vertebral endplate, thoracolumbar fascia, and paraspinal muscle represent anatomically distinct candidate nociceptive niches with different baseline sensory organization and evidence maturity. **(A)** Disc degeneration can permit pathological nociceptive and neurovascular ingrowth into normally sparsely innervated inner regions through mechanisms including NGF–TrkA and macrophage-derived VEGF-A signaling. **(B)** Endplate pathology occurs within a pre-existing intraosseous sensory network; osteoclast-derived Netrin-1, PGE2–EP4–TRPV1 signaling, and Piezo2–CGRP-dependent nerve–endplate feedback provide mechanistic links between osteochondral remodeling and afferent sensitization in experimental models. **(C)** The thoracolumbar fascia contains a pre-existing sensory network that can undergo inflammation-associated afferent redistribution and sensitization. **(D)** Paraspinal muscle exhibits inflammatory–fibrotic pathology and structural remodeling of proprioceptive muscle spindles, whereas persistent nociceptor remodeling remains unproven. The central molecular module denotes overlapping candidate signals rather than proven functional convergence of tissue-specific afferents. The dorsal root ganglia and spinal dorsal horn are therefore depicted as candidate sites of downstream integration; whether different lumbar tissue afferents recruit common neuronal populations remains to be demonstrated. Muscle-spindle remodeling is shown as a separate proprioceptive/sensorimotor branch and is not assumed to contribute directly to nociceptive circuitry. Solid mechanistic arrows indicate experimentally supported links, whereas dashed arrows denote proposed or incompletely established relationships. Evidence-status bars summarize evidence maturity and do not represent formal grading. CGRP, calcitonin gene-related peptide; DRG, dorsal root ganglion; EP4, prostaglandin E receptor 4; IL, interleukin; NF-κB, nuclear factor kappa B; NGF, nerve growth factor; PGE2, prostaglandin E2; RAMP1, receptor activity-modifying protein 1; TRPV1, transient receptor potential vanilloid 1; VEGF-A, vascular endothelial growth factor A.

**Table 1:** Evidence appraisal of tissue-specific peripheral sensory remodeling across candidate nociceptive niches in chronic low back pain.

Nociceptive niche	Dominant sensory phenotype	Human observations	Experimental causality	Pain-related outcomes	Evidence appraisal and key gap
Intervertebral disc	Pathological nociceptive ingrowth into normally sparsely innervated inner disc regions, accompanied by neurovascular remodeling and afferent sensitization.	Deep nerve ingrowth and NGF/TrkA-associated innervation have been demonstrated in painful degenerative human discs (Freemont et al., 1997, 2002).	Degenerative nucleus pulposus promotes sensory neurite growth that is attenuated by NGF blockade; inhibition of macrophage-derived VEGF-A reduces neurovascular remodeling (Yamauchi et al., 2009; Hou et al., 2022).	Human tissue evidence derives from patients with chronic back pain; experimental inhibition of neurovascular mechanisms reduces pain-related behavior (Hou et al., 2022).	Established pathological nociceptive ingrowth. Direct human biomarkers that distinguish an actively nociceptive disc from incidental structural degeneration remain lacking.
Vertebral endplate	Osteochondral sensory remodeling and afferent sensitization, including increased sensory innervation of porous endplate regions.	Human anatomical and cadaveric studies demonstrate intraosseous/endplate innervation but do not, by themselves, establish pain causality (Bailey et al., 2011; Fields et al., 2014).	Osteoclast-derived Netrin-1, PGE2–EP4–TRPV1, and Piezo2–CGRP–RAMP1 pathways have been causally manipulated in experimental models (Ni et al., 2019; Liu et al., 2021; Xu et al., 2026).	Perturbation of these pathways alters mechanical hypersensitivity or pain-related behavior in animal models.	Established mechanistic sensory remodeling and sensitization; causal evidence predominantly preclinical. Direct identification of persistently nociceptive endplate states in humans remains limited.
Thoracolumbar fascia	Sensitization and inflammation-associated remodeling of pre-existing afferents rather than de novo nerve invasion.	Human thoracolumbar fascia is innervated, and experimental stimulation evokes prolonged and spatially extensive pain (Tesarz et al., 2011; Schilder et al., 2014,^2016^; Vogel et al., 2022).	Experimental inflammation causes layer-specific afferent redistribution, increased substance P-positive fibers, and dorsal-horn sensitization in rats (Hoheisel et al., 2015; Hoheisel and Mense, 2015).	Human experimental stimulation produces pain and long-term potentiation-like amplification; clinical hypersensitivity has also been reported.	Established nociceptive capacity; chronic sensory remodeling incompletely defined. Durable afferent remodeling in human CLBP remains unproven.
Paraspinal muscle	Inflammatory–fibrotic pathology with sensory end-organ remodeling; persistent remodeling of nociceptive afferents remains unresolved.	Human studies demonstrate degeneration, inflammation, reduced vascularity, fibrosis, and fatty infiltration, but not persistent nociceptor remodeling (Shahidi et al., 2017; Anderson et al., 2022).	Disc injury induces multifidus muscle-spindle capsule fibrosis in large-animal models, and targeted multifidus activation modifies this structural phenotype (James et al., 2022, 2025). These experiments concern proprioceptive sensory organs rather than nociceptors.	Multifidus stimulation evokes experimental pain; tissue abnormalities show limited clinical associations, but causal nociceptor remodeling has not been established.	Emerging sensory–inflammatory niche. Direct nociceptor remodeling in human disease and the functional sensory consequences of muscle-spindle remodeling remain unresolved.

Evidence is separated into human observations, experimental causality, and pain-related outcomes to distinguish anatomical or molecular associations from causal mechanistic evidence. “Established” indicates a defined sensory phenotype supported by at least two complementary evidence domains, including either direct human evidence or experimental perturbation linked to a pain-related outcome. “Emerging” indicates predominantly indirect or preclinical evidence, absence of direct demonstration of the proposed sensory phenotype, or an unresolved causal relationship with pain. These categories are descriptive and do not constitute formal GRADE assessment. CGRP, calcitonin gene-related peptide; CLBP, chronic low back pain; DRG, dorsal root ganglion; EP4, prostaglandin E receptor 4; NGF, nerve growth factor; PGE2, prostaglandin E2; RAMP1, receptor activity-modifying protein 1; TRPV1, transient receptor potential vanilloid 1; VEGF-A, vascular endothelial growth factor A.

### 1.1 Literature search and evidence appraisal

PubMed/MEDLINE was searched from database inception to 10 September 2026 using combinations of terms for low back pain; intervertebral disc; vertebral endplate or basivertebral nerve; thoracolumbar fascia; multifidus or paraspinal muscle; sensory innervation, nerve ingrowth, nociceptor, dorsal root ganglion, and sensitization. Reference lists were screened for additional original studies. Priority was given to human anatomical or clinical studies and experiments directly assessing sensory remodeling, mechanistic perturbation, or pain-related outcomes; reviews were used mainly for citation tracing. Preprints, unpublished material, and studies without direct sensory or pain relevance were excluded. “Established” denotes a defined sensory phenotype supported by at least two complementary evidence domains—human observation, experimental causality, or pain-related outcome—including either direct human evidence or mechanistic perturbation linked to pain. “Emerging” denotes predominantly indirect or preclinical evidence, an incompletely demonstrated sensory phenotype, or an untested causal relationship with pain. These categories are descriptive and do not constitute formal evidence grading.

## 2 Intervertebral disc: pathological nociceptive ingrowth into a sparsely innervated compartment

Among lumbar tissues, the intervertebral disc provides the clearest example of a nociceptive niche formed through pathological expansion of sensory innervation. In the healthy adult disc, neural fibers are concentrated largely in the outer annulus fibrosus, whereas the inner annulus and nucleus pulposus remain sparsely innervated. Recent anatomical mapping confirms a heterogeneous, multisegmental afferent supply that is predominantly restricted to the outer annulus (Chen L. et al., 2023).

Degeneration disrupts this spatial organization. Human histological studies identified nerve fibers extending into normally poorly innervated inner disc regions in patients with chronic back pain (Freemont et al., 1997). Subsequent analyses linked abnormal innervation to local nerve growth factor (NGF) expression and TrkA-positive fibers, often in close association with ingrowing microvessels (Freemont et al., 2002). Conditioned medium from degenerative human nucleus pulposus also promotes dorsal root ganglion (DRG) neurite extension and substance P expression in vitro, whereas NGF blockade suppresses neurite growth (Yamauchi et al., 2009). These observations support an NGF–TrkA neurotrophic axis through which degeneration may render normally protected inner disc regions permissive to nociceptive ingrowth.

Neural remodeling is closely linked to inflammation and vascular invasion. Vascular endothelial growth factor A (VEGF-A)-producing macrophages promote disc angiogenesis and sensory innervation, whereas inhibition of this macrophage phenotype reduces neurovascular remodeling and degeneration-associated pain (Hou et al., 2022). Disc injury can also increase NGF and TrkA expression, together with CGRP, substance P, interleukin-1β, and interleukin-6, in both injured and adjacent uninjured discs, suggesting that multisegmental afferents and neurogenic inflammation may extend nociceptive signaling beyond the primary lesion (Li et al., 2024).

Changes within the disc are accompanied by sensory neuron plasticity. Disc injury increases oxidative stress and the expression of transient receptor potential vanilloid 1 (TRPV1), CGRP, and substance P in the DRG (Chen et al., 2024), while single-nucleus transcriptomic analysis of experimental chronic discogenic pain identifies persistent changes in pain- and mechanosensation-associated genes, including Scn9a, Piezo2, Fstl1, and Ngfr (Caparaso et al., 2026). Discogenic pain may therefore evolve from local neurotrophic and neurovascular remodeling toward a broader state of persistent peripheral sensory neuron plasticity.

## 3 Vertebral endplate: osteochondral sensory remodeling and vertebrogenic nociception

The vertebral endplate differs fundamentally from the disc because it lies within an already innervated osteochondral and intraosseous compartment. Human anatomical studies demonstrate PGP9.5-positive nerve fibers within the vertebral body and central endplate region, frequently accompanying intraosseous vessels and connecting anatomically with the basivertebral nerve pathway (Bailey et al., 2011). In cadaveric lumbar specimens, endplate pathologies were more frequently and extensively innervated than disc pathologies, although the clinical pain status of these tissues could not be determined (Fields et al., 2014). These findings provide an anatomical substrate for vertebrogenic nociception without implying that every innervated endplate lesion is painful.

Bone remodeling appears to be an important component of this niche. In aged mice and models of lumbar instability, osteoclast activity generates porous endplates and promotes CGRP-positive sensory innervation. Osteoclast-derived Netrin-1 stimulates sensory nerve growth, while elevated prostaglandin E2 (PGE2) contributes to spinal hypersensitivity (Ni et al., 2019). PGE2 acting through prostaglandin E receptor 4 (EP4) further enhances TRPV1-dependent DRG excitability, and pharmacological disruption of this pathway reduces pain-related responses (Liu et al., 2021). Osteochondral remodeling can therefore alter both sensory innervation and afferent excitability.

Recent work adds a mechanically driven nerve–tissue feedback mechanism. Excessive mechanical stress activates Piezo2 in DRG sensory neurons and increases CGRP release. CGRP then acts through receptor activity-modifying protein 1 (RAMP1) on cartilage endplate cells and, together with mechanical stress, activates nuclear factor-κB (NF-κB) signaling, increasing interleukin-1β and interleukin-6 production. These inflammatory signals can, in turn, further sensitize DRG neurons (Xu et al., 2026). Inhibition of Piezo2 or CGRP signaling attenuates pain-related behavior and endplate inflammation in this experimental setting, providing causal support for a bidirectional mechanical–neuroinflammatory loop. Endplate injury can also increase dorsal horn substance P and activate spinal microglial and astrocytic responses, linking peripheral pathology to spinal amplification (Lai et al., 2023).

The endplate can therefore be viewed as an osteochondral sensory niche in which bone remodeling, mechanotransduction, inflammation, and neural plasticity intersect. The key unresolved issue is not whether endplates can be innervated, but which structural endplate abnormalities acquire the capacity to sustain nociceptive signaling.

## 4 Thoracolumbar fascia: sensitization and remodeling of a pre-existing sensory network

The TLF differs from the disc and endplate because it possesses an established sensory network under physiological conditions. Histological studies demonstrate sensory innervation of the TLF in both rats and humans, while quantitative analyses in rats identify CGRP- and substance P-positive free nerve endings consistent with a nociceptive phenotype (Tesarz et al., 2011). Fascial nociception is therefore more plausibly driven by sensitization and remodeling of pre-existing afferents than by de novo neural invasion.

Experimental inflammation provides evidence of such plasticity. In rats, TLF inflammation causes layer-specific redistribution of sensory innervation and significantly increases the density of substance P-positive fibers despite relatively little change in overall innervation density (Hoheisel et al., 2015). Under similar inflammatory conditions, lumbar dorsal horn neurons show increased spontaneous activity and responsiveness, linking altered fascial input to spinal sensitization (Hoheisel and Mense, 2015).

Human experimental studies confirm that the TLF can generate substantial nociceptive input. Hypertonic saline stimulation produces prolonged and spatially extensive pain, while high-frequency electrical stimulation can induce long-term potentiation-like pain amplification (Schilder et al., 2014, 2016). Direct comparisons further show greater pain intensity, duration, and radiation after TLF stimulation than after multifidus stimulation under some experimental conditions (Vogel et al., 2022). Clinical findings are more nuanced. Individuals with myofascial low back pain can exhibit hypersensitivity of both deep fascia and muscle, suggesting that their relative contributions vary across phenotypes rather than following a universally fascia-dominant pattern (Cozacov et al., 2022).

Experimental observations also challenge a strict distinction between “muscle” and “fascial” pain. Sensory characteristics of lumbar delayed-onset soreness more closely resemble experimentally evoked fascial than muscle pain (Brandl et al., 2024). However, these findings do not establish persistent neural remodeling in chronic human CLBP. The TLF is therefore best considered an established nociceptive but incompletely characterized remodeling niche, particularly with respect to durable changes in nociceptor density, molecular phenotype, or spatial organization in human disease.

## 5 Paraspinal muscle: an emerging sensory–inflammatory niche

Paraspinal muscle, particularly the lumbar multifidus, is a less well-defined but potentially important nociceptive niche. Human biopsy and molecular studies identify degeneration, inflammation, reduced vascularity, fibrosis, fatty infiltration, and altered muscle-quality-associated transcriptional programs in lumbar pathology (Shahidi et al., 2017; Anderson et al., 2022; Chen X. et al., 2023). Such abnormalities create an inflammatory–fibrotic microenvironment capable of influencing afferent activity, but they do not establish chronic nociceptor sprouting or phenotypic switching.

The nociceptive capacity of paraspinal muscle is nevertheless well supported experimentally. Hypertonic saline injection into the multifidus evokes local and radiating low back pain, although the magnitude and spatial spread are generally smaller than those produced by comparable TLF stimulation (Vogel et al., 2022). In patients undergoing surgery for lumbar disc herniation, pathological muscle fibers were more prominent at the affected multifidus level, while smaller type I fiber dimensions showed weak associations with greater pain intensity and disability (Purushotham et al., 2024). Whether these structural abnormalities are accompanied by persistent changes in nociceptor density, ion-channel expression, or neuropeptide phenotype remains unresolved.

A distinct sensory component involves muscle spindles and should not be conflated with nociception. In a large-animal model of intervertebral disc degeneration, multifidus muscle spindles developed thicker connective-tissue capsules with increased collagen I and III expression, providing direct evidence of structural remodeling of a proprioceptive sensory organ (James et al., 2022). These structural changes could plausibly influence spindle mechanics, but their effect on sensory transduction was not directly measured. Subsequent targeted multifidus activation reduced spindle capsule thickness and collagen I accumulation after disc injury, indicating that this structural remodeling is at least partly modifiable (James et al., 2025).

Paraspinal muscle may therefore contribute to CLBP through two potentially interacting pathways: nociceptive input arising from an inflammatory–fibrotic muscle environment and structural remodeling of proprioceptive sensory organs. Altered proprioceptive input could, in principle, influence sensorimotor control and spinal loading, although this link remains incompletely demonstrated. Direct evidence of persistent nociceptor remodeling in human CLBP is still lacking, supporting classification of paraspinal muscle as an emerging sensory–inflammatory niche rather than an established nociceptor-remodeling compartment.

## 6 Molecular overlap and candidate sites of sensory-circuit integration

Across lumbar tissues, recurring neurotrophic, inflammatory, neuropeptide, and mechanosensory mediators indicate molecular overlap, but shared mediators do not establish functional convergence of tissue-specific afferents within common sensory circuits. Evidence remains compartment-specific. Disc studies support neurovascular ingrowth and DRG molecular plasticity, whereas endplate models provide causal evidence for osteoclast–sensory signaling and Piezo2–CGRP-dependent feedback (Hou et al., 2022; Ni et al., 2019; Xu et al., 2026). Experimental fascial inflammation alters afferent organization and sensitizes dorsal horn neurons (Hoheisel et al., 2015; Hoheisel and Mense, 2015). Paraspinal muscle studies primarily demonstrate inflammatory–fibrotic pathology and structural remodeling of muscle spindles rather than a defined nociceptor circuit.

The dorsal root ganglion (DRG) is therefore best considered a candidate site of downstream integration and sensory neuron plasticity rather than a proven convergence node. TRPV1 contributes to disc- and endplate-associated nociception through different upstream mechanisms, while Piezo2-dependent mechanotransduction has been demonstrated particularly in experimental endplate pathology (Liu et al., 2021; Chen et al., 2024; Xu et al., 2026). Single-nucleus transcriptomics in experimental discogenic pain further identifies altered *Scn9a*, *Piezo2*, *Fstl1*, and *Ngfr* expression (Caparaso et al., 2026). These findings establish sensory neuron plasticity in specific models, but not recruitment of the same DRG neuronal populations by different lumbar tissues.

Likewise, recurrence of CGRP, substance P, or inflammatory mediators across models indicates molecular overlap rather than circuit-level convergence. Adaptive immune mechanisms may also contribute to persistent sensitization; in a mouse disc-injury model, B-cell germinal-center formation and pronociceptive antibody production accompanied chronic nociceptive sensitization (Wei et al., 2025). Direct tests will require dual-tissue retrograde tracing, tissue-selective stimulation combined with DRG or dorsal horn calcium imaging or electrophysiology, and longitudinal mapping of activated neuronal ensembles. Muscle-spindle remodeling should remain separate from this nociceptive axis: current evidence supports a potential proprioceptive or sensorimotor mechanism unless direct coupling to nociceptive circuitry is demonstrated. The framework therefore predicts potential downstream integration while leaving circuit segregation versus convergence as an experimentally testable question.

## 7 Discussion

### 7.1 Controversies: from a single pain generator to dynamic nociceptive niches

A longstanding question in CLBP is whether persistent pain can be attributed to a single anatomical “pain generator.” The evidence reviewed here favors a dynamic interpretation: disc, endplate, TLF, and paraspinal muscle differ in sensory organization and pathological responses, yet more than one compartment may contribute nociceptive input in the same individual. We therefore propose a dynamic multi-niche model in which tissue contributions vary with disease stage, loading, inflammation, and sensory neuron state. Structural abnormality is not equivalent to an active nociceptive niche; degeneration, Modic-type endplate changes, fascial abnormalities, or muscle fatty infiltration may be incidental rather than dominant pain sources. This distinction is compatible with mechanism-based phenotyping of nociceptive, neuropathic, and nociplastic low back pain (Nijs et al., 2024).

Peripheral drive and central amplification should likewise not be treated as mutually exclusive. Lumbar pathology can accompany DRG plasticity and sensitization-associated spinal changes, but current evidence does not show that spinal amplification necessarily becomes independent of peripheral input. Conversely, identifying a peripheral niche does not exclude nociplastic mechanisms. The framework therefore addresses peripheral nociceptive contributions to CLBP rather than all forms of the disorder.

### 7.2 Current research gaps

Evidence maturity is uneven across tissues. Human histology supports deep nerve ingrowth in degenerative discs, whereas much causal endplate evidence is preclinical. Human experiments establish TLF nociceptive capacity but not durable sensory-fiber remodeling in chronic CLBP. In paraspinal muscle, inflammatory–fibrotic pathology and muscle-spindle remodeling are documented, but persistent nociceptor remodeling remains uncharacterized. Phenotype mismatch is another limitation: cadaveric anatomy, experimental pain in healthy volunteers, surgical tissues, and animal injury models address different questions and should not be treated as interchangeable evidence.

Cross-tissue and longitudinal studies are scarce. Common experimental platforms are needed to distinguish tissue-specific mechanisms from shared downstream responses, while longitudinal designs should separate initiating from maintenance mechanisms. Structural degeneration, imaging abnormalities, sensory signatures, and clinical pain should be measured as distinct variables rather than assumed to be equivalent.

### 7.3 Testable predictions of the dynamic multi-niche model

The framework generates explicit predictions. First, an active niche should show concordance among tissue pathology, a tissue-linked sensory or molecular signature, and the pain phenotype; selective perturbation should reduce pain-related output, whereas incidental degeneration should lack this concordance. Second, if tissue contributions shift over time, longitudinal changes in niche-linked biomarkers, provocation responses, or targeted-intervention responses should track symptoms more closely than static imaging alone. Third, true circuit convergence predicts that selective stimulation or tracing of different lumbar tissues will recruit partially overlapping DRG or dorsal horn neuronal populations; absence of such overlap would support parallel tissue-specific circuits. Muscle-spindle remodeling should be tested against proprioceptive and sensorimotor outcomes rather than assumed to contribute directly to nociception. These predictions allow the model to be supported, refined, or falsified.

### 7.4 Future developments: molecular and epigenomic definition of active niches

Future work should move from abnormal structures toward spatially and molecularly resolved active niches. Single-cell and spatial transcriptomic approaches can map interactions among sensory afferents, immune, vascular, skeletal, and stromal cells. Human spatial transcriptomics has revealed molecular heterogeneity among DRG nociceptors (Tavares-Ferreira et al., 2022), while single-nucleus analysis in experimental discogenic pain identifies persistent sensory neuron reprogramming (Caparaso et al., 2026). Longitudinal tissue and neural profiling could test whether particular molecular states track activation or resolution of individual niches.

Chromatin regulation is an additional hypothesis-generating mechanism for persistence of altered cellular states. Outside the low-back-pain field, nuclear FGF2 regulates bone marrow mesenchymal stromal cell fate through phase-separation-associated rDNA chromatin architecture and super-enhancer-linked genomic organization (Zhang et al., 2025), whereas ANKRD1 maintains a neurogenic mesenchymal stromal cell state by binding neural-gene super-enhancers and preserving open chromatin (Wang et al., 2026). These studies do not establish chromatin regulation as a mechanism of CLBP or lumbar sensory remodeling. However, human DRG chromatin-accessibility profiling demonstrates regulatory heterogeneity involving nociception-related genes in donors without chronic pain (Franco-Enzástiga et al., 2025), providing a more directly relevant rationale for testing whether persistent nociceptive states acquire stable epigenomic features.

The translational goal is to distinguish structural degeneration from biologically active nociception. Molecular imaging, spatial proteomics, and tissue- or blood-derived biomarkers could complement conventional imaging, while prospective perturbation studies could determine whether a molecularly defined niche predicts treatment response. Candidate pathways such as NGF–TrkA, osteoclast–Netrin-1, PGE2–EP4–TRPV1, Piezo2–CGRP signaling, and selected neuroimmune mechanisms should remain tissue- and model-dependent hypotheses until validated in phenotyped human CLBP. The value of the multi-niche framework will ultimately depend on whether it improves causal inference and predicts when, where, and in whom a peripheral tissue is actively sustaining pain.
